# Neurological Associations of COVID-19—Do We Know Enough: A Tertiary Care Hospital Based Study

**DOI:** 10.3389/fneur.2020.588879

**Published:** 2020-11-24

**Authors:** Suman Kushwaha, Vaibhav Seth, Prateek Bapat, KiranGowda R, Monali Chaturvedi, Renu Gupta, Sonali Bhattar, Siddharth Maheshwari, Aldrin Anthony

**Affiliations:** ^1^Department of Neurology, Institute of Human Behaviour and Allied Sciences, New Delhi, India; ^2^Department of Neuroradiology, Institute of Human Behaviour and Allied Sciences, New Delhi, India; ^3^Department of Microbiology, Institute of Human Behaviour and Allied Sciences, New Delhi, India; ^4^Department of Microbiology, Rajiv Gandhi Super Speciality Hospital, New Delhi, India

**Keywords:** SARS-CoV-2, neurological manifestation, stroke, meningoencephalitis, GBS, neurotrophic potential

## Abstract

The neurotrophic potential of SARS-CoV-2 virus is manifesting as various neurological disorders in the present pandemic. Nervous system involvement can be due to the direct action of the virus on the brain tissue or due to an indirect action through the activation of immune-mediated mechanisms. This study will discuss the detailed systematically evaluated clinical profile and relevant investigations and outcome of 14 laboratory confirmed SARS-CoV-2 positive patients presenting with neurological signs and symptoms. The patients were further categorized into confirmed, probable, and possible neurological associations. The probable association was found in meningoencephalitis (*n* = 4), stroke (*n* = 2), Guillain-Barré syndrome (*n* = 1), and anosmia (*n* = 1). The other six patients had coexisting neurological diseases with SARS-CoV-2. One patient with a large artery stroke succumbed to the illness due to respiratory complication. Memory impairment as a sequela is present during follow up of one encephalitis patient. Presently the early recognition and diagnosis of neurological manifestations remains a challenge for clinicians as the SARS-CoV-2 related neurological manifestations are in evolution. A long-term correlation study of clinical profile, radiological and laboratory investigations, along with neuropathological studies is needed to further understand the pathophysiology behind the SARS-CoV-2 neurological manifestations. Further understanding will facilitate timely recognition, therapeutic intervention, and possible prevention of long-term sequalae.

## Introduction

The pandemic due to COVID-19 is growing exponentially worldwide after first being notified from Wuhan, China, in December 2019. The virus that causes COVID-19 is designated as severe acute respiratory syndrome coronavirus 2 (SARS-CoV-2). Primarily this virus affects the respiratory system and thereby initiates the cascade of sequences that involves multiple organs. There are evidences of involvement of other systems like nervous system, cardiovascular system, and gastrointestinal system, which are a matter of scientific study and research. Six months into this pandemic, the understanding and recognition of neurological manifestations and complications has increased as evident from the cases series and reports in literature (1). There are reports of COVID-19 patients presenting with various neurological manifestations, and in others there is exacerbation of underlying neurological illness. The neurological presentations include dizziness, headache, anosmia, ageusia, seizure, meningitis, encephalitis, stroke, and acute inflammatory demyelinating polyneuropathy.

The neurotrophic potential of SARS-CoV-2 is being recognized and well-established in some studies by detection of SARS-CoV-2 in CSF by RT-PCR and its culture from brain tissue biopsy (2–4). The limited available data on neurological manifestations raises important concerns regarding the extent of nervous system involvement and the pathogenesis causing these manifestations. There is need for an early recognition and diagnosis as shown in a few studies (5).

In our clinical practice during the pandemic we have systematically evaluated and investigated patients for evidence of this new emergent virus and its associated neurological manifestations. This clinical study will facilitate in understanding the connection between SARS-CoV-2 and different clinical presentation. Early identification of the various probable neurological manifestations of COVID-19 will be instrumental in deciding the available therapeutic options and reducing the possible long-term complications.

## Materials and Methods

A cross-sectional study came from April 2020 to July 2020 at Institute of Human Behavior and Allied Sciences, a tertiary care neuropsychiatry center in North India.

This Institute caters to acute and chronic neurology and psychiatry patients in emergency and outpatient departments. The study was conducted in Department of Neurology.

All the admitted neurological patients during the study period had been evaluated for neurological symptoms and signs and carefully assessed and investigated for any atypical neurological finding. Patients were also monitored for the presence of influenza like illness (ILI) considering the current pandemic.

Routine hematological (Complete Blood Count, ESR), biochemical parameters (blood sugar, LFT, KFT, electrolytes, thyroid functions, CRP, serum ferritin) were done. X ray chest, ultrasound abdomen, and neuroimaging–brain MRI and CT head was done in all patients.

Autoimmune Profile (NMDA, LGi1, AMPA, CASPR-2) was done in selected patients.

CSF examination was selectively done for cytology and biochemistry.

Neuroviral panel (herpes simplex virus1 & 2, mumps virus, varicella-zoster virus, enterovirus, parechovirus) was done in all CSF samples of patients suspected of having meningoencephalitis. CSF PCR for SARS-CoV-2 was done in all patients of meningoencephalitis.

The neurological diagnosis was made after evaluating the clinical history, examination, and relevant investigations. The patients were critically evaluated and investigated for atypical neurological presentation.

Patients with atypical neurological presentation and with influenza like illness (ILI) in the recent past or during hospitalization were categorized into suspected COVID-19.

The real-time reverse-transcription polymerase chain reaction assay using a SARS-CoV-2 nucleic acid was done from oral and nasopharyngeal sample in all patients, from CSF in selected patients by the below mentioned method.

Nucleic acid extraction was done using chemagic Viral DNA/RNA 300 Kit H96 in Chemagic 360 instrument from Perkin Elmer as per the manufacturer's instruction. The sample processing was carried out in BSL-2 following standard precautions. An amount of 315 μl of lysis buffer was added to 300 μl of sample volume for extraction. Internal reference of 5 μl as provided by the FOSUN COVID-19 PCR kit was added to each sample to monitor the efficiency of sample preparation and to differentiate between true negative and false negative. A qualitative PCR test was done subsequently with the FOSUN COVID-19 PCR detection kit using primer-probe mixture for detection of N gene, E gene, and ORF1ab gene. The results were interpreted as per the kit insert.

The laboratory confirmed cases of COVID-19 with neurological manifestations are included in study. The demographic, clinical, laboratory investigations, follow up, and outcome of the patients is presented.

We obtained written consent from patients or their relatives for publication of the data including images and any identifiable data that might reveal a patient's identity.

## Results

A total of 358 patients with neurological diagnosis were admitted in the hospital during the study period. Out of these, 69 cases were suspected of having COVID-19 disease and there were 14 laboratory confirmed cases.

The detailed demographic, clinical profile, investigations, and outcome has been summarized in Table 1.

**Table 1:** Clinical features, investigations, treatment, and outcome of 14 positive SARS-CoV-2 cases.

**S. No**.	**Patient demographic**	**Neurological presentation**	**Associated COVID-19 symptoms**	**Positive SARS-CoV-2 test type**	**Relevant blood investigations and radiologic findings**	**Neurological investigations (CSF findings, neuroimaging)**	**Treatment and outcome**
		**Cerebrovascular accidents**					
1	55 yr F	Patient presented with left hemiparesis with global aphasia.	Two weeks after the stroke developed fever with dyspnoea.	Upper Respiratory swab PCR	Increased total cell count with neutrophilic leucocytosis. Mildly deranged transaminases with deranged INR (2.54). CRP was raised. D-dimer was raised. Chest X ray showed an opaque left hemi thorax suggestive of a collapse/consolidation.	CT brain showed right malignant MCA infarct. MRA showed MCA main stem occlusion	Treated conservatively for stroke. After development of COVID-19 symptoms required intubation and mechanical ventilator. Died within 2 days of diagnosis of COVID-19.
2	70 yr F	Patient presented with sudden onset left hemiparesis (lower limb more than upper limb), NIHSS 6 at the time of admission with a window period of 3.5 h.	Cough and sore throat at the time of admission.	Upper Respiratory swab PCR	Normal blood counts and other parameters. CRP was raised. Ferritin Normal.	Infarct in right centrum semiovale. Left CCA showing 30% stenosis.	Was thrombolysed with alteplase. Post-thrombolysis her NIHSS improved from 6 to 4. She was treated with azithromycin, hydroxychloroquine, and was discharged on day 15 post-admission.
		**Meningoencephalitis**					
3	15 yr M	Patient presented with fever and headache from 5 days prior to admission.	Sore throat, diarrhea, and fever 5 days prior to admission.	Upper Respiratory swab PCR positivity, negative CSF PCR	Routine investigations were normal.	CSF study revealed an opening pressure of 30 cm of water, 12 cells (60% lymphocytes, and 40% neutrophils) with normal sugar, protein levels. Negative culture and Virology results with a negative TB PCR. MRI brain was normal.	Empirically started on acyclovir but had disabling headache. Put on dexamethasone, topiramate, acetazolamide. Required a repeat lumbar puncture for therapeutic purpose. Discharged on tapering dose of dexamethasone, acetazolamide, and topiramate. One month into follow up patient is symptom free and not on any medication.
4	35 yr F	Presented with new onset focal seizures with impaired awareness, acute onset memory impairment.	Fever 7 days prior to presentation.	Upper Respiratory swab PCR positivity, negative CSF PCR	Routine investigations normal.	CSF study 100 cells with 90% lymphocytes and mildly raised protein (56mg/dl). Negative cultures and virology panel. MRI showed T2/Flair hyperintensity in left temporo-occipital lobe, hippocampus with diffusion restriction, and right frontal periventricular white matter T2 flair hyperintensity (Figure 1). EEG showed generalized slowing.	Empirically started on acyclovir and levetiracetam. Then put on dexamethasone. Discharged after 14 days of inpatient stay with a diagnosis of probable COVID-19 encephalitis.
5	38 yr M	Presented with fever, headache, altered behavior.	Fever 5 days prior to admission.	Upper Respiratory swab PCR positivity, negative CSF PCR	Routine investigations normal.	Lumbar puncture showed 200 cells with 90% lymphocytes with increased protein. Negative cultures and virology pattern. Negative TB PCR. MRI brain with contrast normal.	Treated empirically with acyclovir but gradual improvement in symptoms, no other treatment given.
6	23 yr M	Presented with headache, fever, altered sensorium.	Fever, myalgia, vomiting, abdominal pain five days prior to admission.	Upper Respiratory swab PCR positivity, negative CSF PCR	Normal counts. Deranged liver function Tests, hyponatremia. CXR showed opacities (Figure 2).	CSF showed 94 cells 80% neutrophils and normal sugar and protein. MRI brain normal/CT Normal. Negative culture and viral serology. TB PCR negative.	Treated with anti tubercular drugs, acyclovir and dexamethasone.
		**Other neurological diseases with COVID-19**					
7	70 yr F	Patient diagnosed case of tubercular meningitis presented with altered sensorium.	Fever Shortness of breath	Upper Respiratory swab PCR. Initial test was Negative	Normal counts with hyponatremia. Rest investigations within normal limit. CT chest showed consolidation in bilateral upper zone and right lower zone.	CSF study showed 140 cells with 90% lymphocytes with normal sugar and increased protein (112 mg/dl). Neuroimaging consistent with TBM with hydrocephalus.	Treated with dexamethasone, anti-tubercular drugs, mannitol, and acetazolamide. She was referred for neurosurgical intervention.
8	25 yr F	Diagnosed case of Tubercular Meningitis with CNS Tuberculoma on treatment presented with status epilepticus.	Fever, Myalgia, Dyspnea 4 days prior to admission.	Upper Respiratory swab PCR	Neutrophilic leucocytosis with hypokalemia, CXR showing right lower zone opacities.	MRI brain with contrast suggestive of Tuberculoma. EEG suggestive of generalized epileptiform discharges. CSF normal study.	Treated for status epilepticus, Anti tubercular drugs, recovered and discharged.
9	15 yr F	Seizures and myoclonus	Asymptomatic	Upper Respiratory swab PCR	Normal investigations.	CSF showed 2 cells with normal sugar and protein. EEG showed slow periodic 2-3Hz discharges. CSF IgG positive for measles antibody	Diagnosed as SSPE - Treated with valproate, levetiracetam. She was asymptomatic. Discharged after monitoring.
10	53 yr M	Presented with status epilepticus and altered mental status.	Asymptomatic	Upper Respiratory swab PCR	Increased counts. Deranged Liver function test. CXR showed Bilateral middle zone opacities.	Gliosis in left fronto parietal lobes. CSF normal study.	Treated for status epilepticus with IV antibiotics and hydroxychloroquine, recovered well and discharged.
11	45 yr M	Diabetic patient presented with right eye ptosis, complete ophthalmoplegia, anosmia, ageusia with headache.	Fever and running nose 10 days prior to admission.	Upper Respiratory swab PCR positivity, negative CSF PCR	Leucocytosis with other normal blood parameters.	Neuroimaging revealed right side cavernous sinus thrombosis with pansinusitis. CSF study showed 35 cells with 90% lymphocytes and normal sugar and protein. Negative for culture and virology. TB-PCR negative.	Treated with IV antibiotics and IV amphoterecin B on suspicion of fungal cavernous sinus thrombosis.
12	48 yr F	Diabetic patient presented with altered sensorium and non-convulsive status.	Asymptomatic	Upper Respiratory swab PCR	Leucocytosis with raised blood sugar and serum osmolality. CXR was normal.	Neuroimaging showed bilateral caudate hyperdensities with hypodensity in left basal ganglia. EEG showing generalized epileptiform discharges. CSF study was normal.	On treatment with IV anti epileptics, insulin infusion.
13	65 yr M	Peripheral nervous system manifestationPresented with paraparesis with progressing weakness to upper limb and dysphagia.	Fever, ageusia five days prior to presentation. Cough present at the time of admission.	Upper Respiratory swab PCR	Neutrophilic Leucocytosis with Thrombocytosis. Hyponatremia. CXR Normal.	Demyelination with secondary axonal changes in nerve conduction studies.	On Intravenous immunoglobulin.
14	30 yr M	Loss of smell and taste.	Asymptomatic	Upper Respiratory swab PCR	Normal	Normal	Isolation and hydroxychloroquine for 5 days.

Among 14 positive SARS-CoV-2 infection patients, there were seven male and female patients, respectively. Median age at onset of symptoms was 41.5 years (range 15–70). The presenting neurological symptoms were altered sensorium (*n* = 6), headache (*n* = 4), seizures (*n* = 5), limb weakness (*n* = 3), anosmia (*n* = 2), while ophthalmoplegia and memory impairment occurred in one patient, respectively.

Influenza like illness (ILI) was present in 10 patients. Nine had fever; sore throat and shortness of breath were present in two patients, diarrhea and myalgia were seen in one patient, respectively. The ILI symptoms were present in seven patients on an average of 1 week prior to admission. One patient developed ILI on admission while other two developed these symptoms after 7–14 days of hospitalization. Four patients were asymptomatic.

Abnormal laboratory parameters included leucocytosis (*n* = 6), hyperglycemia (*n* = 1), deranged INR (*n* = 1), and hyponatremia (n=3). Complete blood counts were normal in eight patients. CRP was raised in two patients. D dimer was raised in one patient. Serum ferritin was found to be normal in all patients.

CSF was abnormal in four patients. Pleocytosis was a significant finding. High opening pressure was seen in one patient. Neuroviral panel for other virus was negative in all patients. Chest X ray was abnormal in four patients. Neuroimaging was normal in six patients. EEG abnormality were seen in four patients.

Patients were categorized into probable SARS-CoV-2 meningoencephalitis in four, stroke associated SARS-CoV-2 in two, GBS in one, and anosmia with ageusia in one. The other six patients had coexisting neurological disease with SARS-CoV-2 infection.

There was mortality in one patient with large artery stroke due to respiratory involvement on day 21 after admission. Eleven patients were discharged who are doing well on follow up with no complications, except one patient who has memory impairment, while two are still admitted.

## Discussion

The neurological manifestations are now being increasingly recognized and reported in different case series from the centers exclusively managing COVID-19 patients. This clinical cross-sectional study presents the different probable neurological manifestations of SARS CoV-2 from a tertiary care neuropsychiatry center. The patients are primarily presenting to us with neurological signs and symptoms attributing to the known neurological disorders. Amid the ongoing pandemic, we have carefully assessed all the patients for the typical COVID-19 symptoms.

The great heterogeneity of neurological presentation for individual patients with viral illnesses has been witnessed in past pandemics. The experience with other earlier pandemics of respiratory pathogens SARS, MERS, and H1N1 influenza and the neurological complications associated with them has given an insight into the present pandemic (6, 7).

The spectrum of neurological presentations has a wide array as reported in recent literature. Anosmia, ageusia, encephalitis, post-infectious acute disseminated encephalomyelitis, cerebrovascular accidents, and Guillain-Barré syndrome are common among the reported manifestations (8). It is important to understand the timing and relation of neurological manifestations and complications associated with SARS-CoV-2, a new emergent virus.

Different mechanisms have been proposed for the entry of the virus into the brain in research studies. Hyposmia and anosmia, the common symptoms, have been explained by the entry of SARS-CoV-2 into brain tissues via dissemination and spread from the cribriform plate, which is in close proximity to the olfactory bulb (9). This neurotrophic virus causes damage by a surge of inflammatory cytokines, mainly Interleukin-6.

Initially, in the largest retrospective study of 1,099 patients with laboratory-confirmed COVID-19 in China, Guan et al. reported respiratory and generalized symptoms as the presenting complaints. The definite neurological symptoms were not reported in this study (10). Later in the recent study from Wuhan, it has been described that 74% of COVID-19 patients can develop neurological symptoms in addition to common respiratory symptoms. Impaired consciousness and stroke were two common symptoms reported in critically ill patients (11).

Recently, a cross-specialty surveillance study from UK reported 62% of patients presented with stroke. Altered mental status was the second most common presentation, comprising encephalopathy or encephalitis and primary psychiatric diagnoses, often occurring in younger patients (12). The neuropsychiatry presentation has also been reported.

In our single center study, 14 patients have fulfilled the WHO confirmed case definition of COVID-19 (13). The detailed clinical characteristics of the 14 patients has been described in Table 1. Accordingly the patients were primarily categorized into probable and possible neurological manifestations due to SARS-CoV-2.

In probable SARS-CoV-2 meningoencephalitis patients, altered sensorium, irritability, and headache were present in all patients. These patients were relatively young (age range 14–50 years). Contrary to our study, eight adults patients aged 24–78 years (median 62 IQR 40–70), four women and men each, have been described with encephalitis associated with COVID-19 (14). Patient 3 has presented with acute onset disabling headache. Mao et al. have reported headache and encephalopathy in 40% of patients in their cohort (15). The brain imaging was unremarkable. His CSF examination had high opening CSF pressure of 30 cm of water. The CSF cytology and biochemical parameters were normal. Subsequently he required repeat therapeutic lumbar puncture as his headache was resistant to other therapeutic options. He was given steroids for 2 weeks and was symptom free on follow up. There is a report of steroid responsive encephalitis in coronavirus disease (16). Raised opening pressure has been described in a patient of COVID-19 related encephalitis by Moriguchi et al. (14).

CSF pleocytosis was a predominant finding indicating brain inflammation in patients with meningoencephalitis. The neuroviral panel was negative for all patients. CSF PCR for SARS-CoV-2 done in four patients was found to be negative. Recently the presence of SARS-CoV-2 RNA in the cerebrospinal fluid has been detected by genome sequencing in a patient with clinically proved meningoencephalitis in Japan (14).

In one study of 183 hospitalized children with clinically suspected acute encephalitis, 22 (12%) had coronavirus infection, the type was not specified by detection of anti-CoVIgM (17). Neuroimaging plays an important role in diagnosing meningoencephalitis.

**Figure 1:**
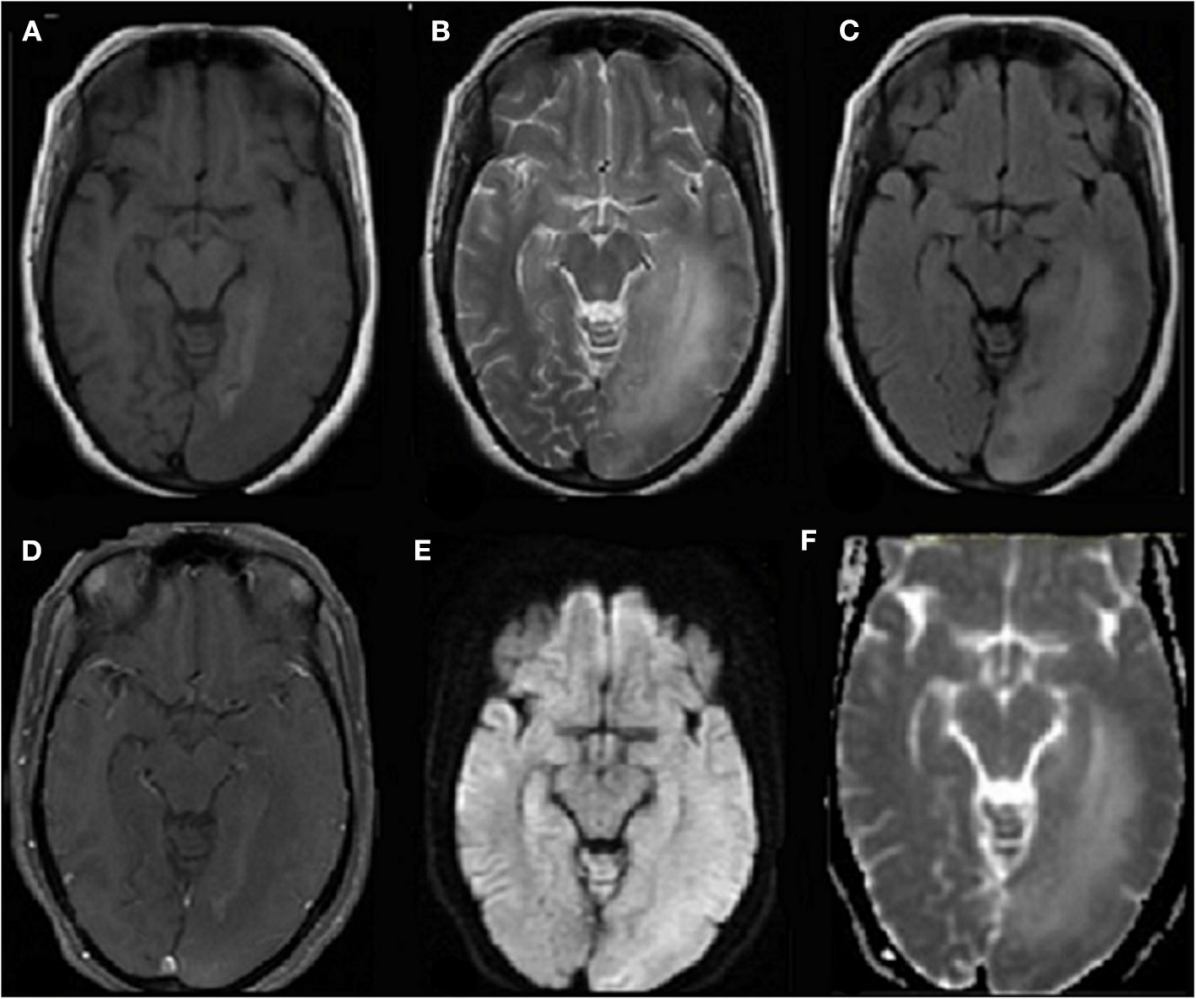
Axial T1 weighted **(A)** MR image shows hypointensity in left parieto-occipital region in subcortical and deep white matter with overlying sulcal effacement and corresponding hyperintensity on T2 weighted image **(B)** and FLAIR **(C)** images. No associated contrast enhancement seen on contrast-enhanced T1-weighted MR image **(D)** with no restricted diffusion on diffusion-weighted MR image **(E)**, and apparent diffusion coefficient (ADC) map **(F)**.

Figure 1 of patient 4 who presented with seizures and altered sensorium is consistent with meningoencephalitis. On follow up after 3 weeks, she has memory impairment as a sequela.

Poyiadji et al. described a report where haemorrhagic ring enhancing lesions consistent with acute necrotizing encephalitis were seen in the bilateral thalami, medial temporal lobes, and sub-insular regions on brain MRI (18). They have proposed that the virus does not directly invade the blood-brain-barrier and acute necrotizing encephalitis is caused by SARS-CoV-2 via cytokine storm (18). In the French series of 58 intensive care patients with COVID-19, the neurological complications were present in 49 (84%) and 40 (69%) had encephalopathy while 39 (67%) had cortico-spinal tract signs. MRI findings in 13 patients showed leptomeningeal enhancement and eight patients had acute ischaemic change (5).

According to the provisional case definition for the association of COVID-19 with neurological disease, the probable case is defined as SARS-CoV-2 detected in respiratory or other non-CNS sample, or evidence of SARS-CoV-2 specific antibody in serum indicating acute infection and no other explanatory pathogen or cause found (1, 13). Accordingly our four cases are categorized into probable meningoencephalitis due to SARS CoV-2 infection.

The possibility of stroke associated with COVID-19 has increased as reported from publications from around the world. In the pooled analysis of four studies on cerebrovascular disease (CVD) with COVID-19, it is concluded that there is 2.5-fold increase in odds of severe COVID-19 illness with a history of stroke and trend toward increased mortality rate (19). The incidence of ~2% is being reported for CVD among patients infected with SARS-CoV-2 admitted to the hospital (11).

SARS-CoV-2 infection is associated with hypercoagulable state and the depletion of angiotensin-converting enzyme 2 (ACE2) that results in tissue damage, including stroke. The binding of SARS-CoV-2 with ACE2 (a cardio-cerebro vascular factor) damages ACE2 and can lead to strokes. The cytokinin related injury also plays a role in causing stroke (20, 21).

There is a predilection for large vessel involvement and male preponderance. Avula et al. reported four patients who initially presented with computed tomography (CT) proven stroke and later tested positive for COVID-19 (22). Patient 1, admitted with right middle cerebral artery infarct, was managed conservatively. She developed fever and breathlessness and was found to be positive for SARS-CoV-2 on day 14 and succumbed to her illness due to respiratory insufficiency. Her X-ray showed an atypical finding of unilateral consolidation and collapse. Both of our stroke patients do not have the conventional risk factors for stroke. Li et al. has described that the median durations from first symptoms of COVID-19 to cerebrovascular disease was 10 days (23).

In the case series from Turkey, four patients with COVID-19 were reported to be simultaneously accompanied by acute ischemic stroke. The average time from COVID-19 symptom onset to the diagnosis of stroke was 2 days in this series (24).

Patient 2 presented in a late window period with NIHSS score of 6. CT head scan was normal. She was thrombolyzed with alteplase. She had a developed fever the next day. Her nasopharyngeal swab PCR was positive for SARS-CoV-2. Her NIHSS improved to 4 in 2 days. Her post-thrombolysis CT scan showed hypodensity in right centrum semiovale. She was managed with antiplatelets and neurorehabilitation.

Intravenous thrombolysis in acute stroke during this pandemic is a bigger challenge. The patients are reaching emergency care late due to several reasons. At large patients with minor strokes are also avoiding attending hospitals for the fear of infection. There is possibility of neurological symptoms being masked by the infection itself, or a delay in access to neuroimaging or revascularization techniques could be responsible as reported by Oxley et al. (25).

The single center retrospective study describes 13 of 221 patients diagnosed with SARS-CoV-2 virus to have ischemic stroke (23). Thrombocytopenia with elevated D-dimer and C-reactive protein in severe COVID-19 and stroke are consistent with a virus-associated microangiopathic process. Inflammatory response along with coagulopathies play an important role in the COVID-19 related stroke. The inflammatory marker, CRP levels were raised in both the patients while D-Dimer was raised in one patient with large artery thrombosis. The treatment comprises of anticoagulation with low-molecular-weight heparin for patients with COVID-19, to reduce the risk of thrombotic disease (26).

Hyposmia, anosmia, and dysgeusia are commonly reported symptoms seen in COVID-19 pausisymptomatic or asymptomatic patients. In a retrospective study by Klopfenstein et al. on analysis of patients with anosmia, he concluded that 47% (54 out of 114) of COVID-19 patients reported anosmia (27). Anosmia began 4.4 [±1.9 (1–8)] days after the onset of infection and the mean duration of anosmia was 8.9 [±6.3 (1–21)] days (27).

Patient 14 had only subacute onset of anosmia and ageusia for around 2 weeks. He was followed up in isolation for development of any other symptoms. No further symptoms developed and he was SARS-CoV-2 negative after 15 days. This is contrary to a patient reported by Eliezer et al. who reported a woman in her 40s presented with hyposmia with a history of dry cough along with headache and generalized fatigue a few days before presentation (28).

The pathophysiologic mechanism underlying the smell and taste impairment needs to be understood; the neurotrophic potential of the SARS-CoV-2 virus is known and it is hypothesized that the virus spread through olfactory bulbs into the central nervous system (29). Screening for SARS-CoV-2 should be done in patients presenting with isolated anosmia.

Neuromuscular disorder has been reported with SARS-CoV-2 by Tsai et al. (30). Guillain-Barré syndrome is an acute polyradiculopathy characterized by rapidly progressive, symmetrical limb weakness, areflexia on examination, sensory symptoms, and facial weakness in some patients. Patient 13 had anosmia and ageusia 5 days prior to the onset of classic symptom of GBS. He developed fever and sore throat on the day of admission. Electrodiagnostic findings confirmed demyelinating neuropathy. Besides the typical GBS, Miller Fisher variant of Guillain-Barré syndrome with ophthalmoplegia, ataxia, and areflexia is also being reported (31, 32).

The common antecedent infections related with GBS are Campylobacter jejuni, Zika virus, and influenza virus (33–35).The SARS-CoV-2 infection stimulates inflammatory cells and produces various inflammatory cytokines and, as a result, it creates immune-mediated damage (36). To our understanding, GBS being an autoimmune disorder, the hypothesis for it as a manifestation of COVID-19 remains unclear.

In our clinical practice we have seen the concomitant infection of tubercular meningitis and SARS-CoV-2 in Patients 7 and 8. The tubercular meningitis is diagnosed at the presentation and simultaneously she was positive for SARS-Co-V-2. Another 6 months follow up stable case of tubercular meningitis with tuberculoma presented in status epilepticus. She was asymptomatic and found to be SARS-CoV-2 positive on day 2 of hospitalization. There are reports wherein the new onset of seizure or status epilepticus can be a presenting symptom of COVID-19 (37). To explore the association of tuberculosis/tubercular meningitis and COVID-19 the global tuberculosis network cohort study is underway. The preliminary analysis suggests that 38.8% of patients had COVID-19 while on antitubercular treatment. There is limited or no protection against COVID-19 in patients of tuberculosis (38).

**Figure 2:**
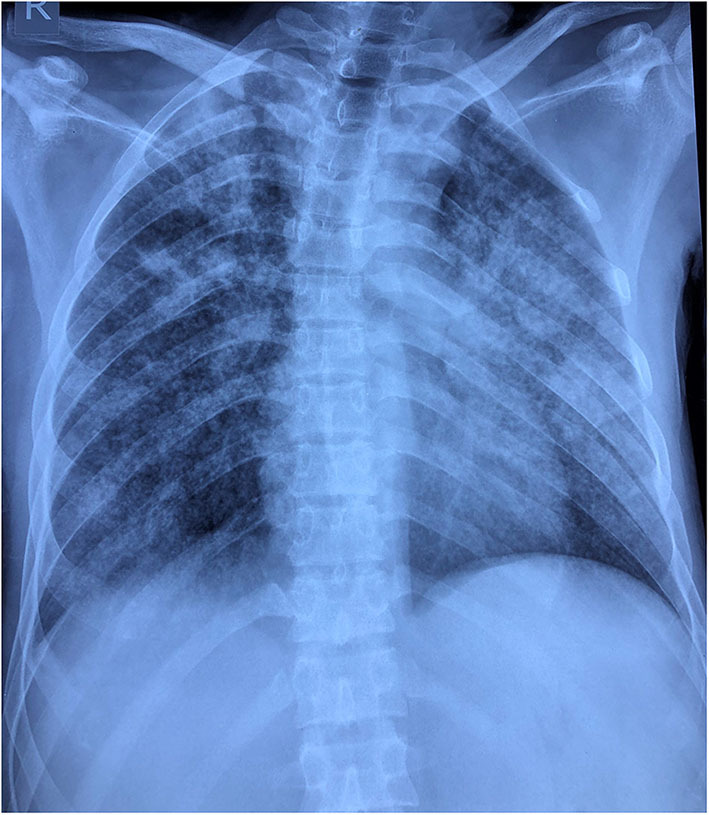
Chest radiograph reveals multifocal patchy peripheral areas of air space opacification scattered in right lung field with right upper zone predominance. Similar confluent opacities with ground glass shadows are evident in the left middle and lower zone with relative sparing of left upper zone.

Patient 6 presented with acute onset encephalopathy. The CSF showed mild pleocytosis with normal sugar and protein, TB PCR and neuroviral panel were negative. Contrast brain MRI was normal. His chest radiograph showed opacities (Figure 2). Tubercular meningitis as a cause of encephalopathy was ruled out. The diagnosis of pulmonary tuberculosis was particularly challenging in this patient in presence of overlapping SARS-CoV-2 symptoms and positive PCR. CT chest was not possible as our hospital does not have a dedicated CT facility for COVID-19 patients. He was started on anti-tubercular drug along with other supportive treatments. In the tuberculosis endemic country of India, during the current COVID-19 pandemic it is challenging to differentiate the acute meningoencephalitis presentation of SARS-CoV-2 from tubercular meningoencephalitis.

An observational study has explored the relationship between MTB infection and COVID-19 pneumonia, suggesting that individuals with latent or active TB may be more susceptible to SARS-CoV-2 infection, and that COVID-19 disease progression may be more rapid and severe (39). A chronic respiratory disease like pulmonary tuberculosis is susceptible to this virus. The treatment for both coexistent diseases should be tailored accordingly, as use of an immunosuppressant will exacerbate tuberculosis.

There is evidence that SARS-CoV-2 virus may penetrate the brainstem, aggravating respiratory impairment (40). Seizures in severe/end-stage disease are likely to be due to COVID-19 related hypoxia, encephalopathy, or encephalitis rather than lowered seizure threshold in susceptible individuals with pre-existent neurologic disease.

There is limited information in literature that suggests that individuals with epilepsy are more likely to be infected by the virus. Giovanni et al. reported worsening of seizures in 18% of people with epilepsy (PwE) during the COVID-19 period, mostly seen in patients who were having poor sleep quality and on multiple antiepileptics. PwE were not found to be at increased risk of acquiring SARS-CoV-2 infection (41). New onset seizures and status epilepticus has been described as a presentation of COVID-19 (42). Musolino et al. investigated preliminary COVID-19 findings and found one out of 10 infected children with seizures, while others presented predominantly with fever, cough, and diarrhea (43).

In our study, five patients presented with seizures and were concomitantly diagnosed with SARS-CoV-2. Patient 9 with history of 1 week duration of myoclonic jerks and generalized seizures was diagnosed with subacute sclerosing panencephalitis (SSPE). Her CSF examination showed 2 cells with normal sugar and protein. CSF IgG measles for antibody was positive. EEG showed slow periodic 2–3 Hz discharges. Patient 10 with post-traumatic seizure disorder, who was well-controlled for 2 years, presented in status epilepticus.

Diabetic patients are considered to be at risk for SARS-CoV-2 infection. Patient 12, a middle aged female with uncontrolled diabetes mellitus, presented in non-convulsive status epilepticus. She was asymptomatic for COVID-19 symptoms and was in non-ketotic hyperosmolar state. Patient 11 presented with right eye ptosis, complete ophthalmoplegia, anosmia, and ageusia with headache. His neuroimaging revealed cavernous sinus thrombosis. In diabetes, due to hyperglycaemic state there is high risk of acquiring infection and progression to severe-stage of COVID-19 leading to immune dysfunction (44). The patients with diabetes are vulnerable to nosocomial infection, which can deteriorate their general condition and aggravate COVID-19 symptoms (45). In this patient we have suspected fungal infection causing cavernous sinus thrombosis due to complicated pan sinusitis with bony destruction on brain imaging.

The presentation of patients with seizures raises the question of whether epilepsy patients are more prone to SARS-CoV-2 infection. COVID-19 may exacerbate epileptic seizures from associated systemic effects not directly related to SARS-CoV-2 CNS infection. The reasonable current understanding remains that in some patients with COVID-19, seizures develop as a consequence of hypoxia, metabolic derangements, organ failure, or even cerebral damage. The association of COVID-19 with the patients of epilepsy needs to be explored further in well-planned studies.

This study gives an insight into the neurological associations of SARS-CoV-2, the new emergent virus in the current pandemic. The infection itself might be a source of neurological manifestations, and patients with neurological disorders might be at risk of the most severe complications of this infection. The effect of SARS-CoV-2 on pulmonary tuberculosis and tubercular meningitis in endemic areas needs to be closely monitored for increased risk of occurrence and its appropriate management.

Our limitations include the spectrum of neurological manifestations from a single center study may not be complete. The long term follow up for possible sequalae is essential to elucidate the pathophysiological mechanism.

Absent of the dedicated CT facility for COVID-19 patients, the respiratory involvement at the early stage may have been missed.

As we are still evolving with our knowledge in the pandemic, identification and reporting of neurological manifestations of SARS-CoV-2 will help in formulating the guidelines and protocol for early diagnosis and management.

## Data Availability Statement

The raw data supporting the conclusions of this article will be made available by the authors, without undue reservation.

## Ethics Statement

Ethical review and approval was not required for the study on human participants in accordance with the local legislation and institutional requirements. Written informed consent to participate in this study was provided by the participants' legal guardian/next of kin. Written informed consent was obtained from the minor(s)' legal guardian/next of kin for the publication of any potentially identifiable images or data included in this article.

## Author Contributions

VS, PB, and KR were involved in patient management and care. SK, VS, PB, and KR summarized and conceptualized the data and drafted the initial manuscript. SK, VS, SM, and AA revised the final manuscript. RG and SB collected the laboratory data including RT PCR for COVID-19. Radiological images have been described by MC. All authors agreed upon the final form of the manuscript before submission.

## Conflict of Interest

The authors declare that the research was conducted in the absence of any commercial or financial relationships that could be construed as a potential conflict of interest.
